# Exploring the impact of internet use on cognitive abilities in the older adults: evidence from the CHARLS 2020 database

**DOI:** 10.3389/fpubh.2025.1510418

**Published:** 2025-02-18

**Authors:** Haojin Jiao, Zehui Guo, Jiafan Sun, Ke Wang, Jingqi Yang

**Affiliations:** ^1^School of Modern Post, Xi'an University of Posts and Telecommunications, Xi'an, China; ^2^School of Marxism, Yan'an University, Yan'an, China; ^3^School of Economics and Management, Xi'an University of Posts and Telecommunications, Xi'an, China; ^4^Department of Cardiovascular Medicine, Jiangxi Provincial People’s Hospital, The First Affiliated Hospital of Nanchang Medical College, Nanchang, China

**Keywords:** cognition, internet access, older adults, CHARLS 2020, cognitive dysfunction, sociodemographic factors

## Abstract

**Introduction:**

The rapid aging of the global population has increased the prevalence of cognitive impairments, presenting significant challenges for healthcare systems. This study examines the potential protective role of internet use against cognitive decline among older adults in China.

**Methods:**

Utilizing the China Health and Retirement Longitudinal Study (CHARLS 2020) data, this research analyzed a sample of 7,142 Chinese adults aged 60 and above. The study employed correlation analysis and hierarchical regression to explore the relationship between various dimensions of internet use and cognitive function.

**Results:**

Results indicate a significant positive correlation between internet use and cognitive performance, suggesting that digital engagement may serve as a protective factor against cognitive decline. Additionally, the study reveals that socio-demographic factors such as age, education level, and urban or rural residency modulate this relationship.

**Discussion:**

The findings underscore the importance of digital inclusivity for enhancing cognitive health among older adults. This study discusses the implications of increasing internet accessibility and provides recommendations for public policy to foster a digitally inclusive society that supports the cognitive health of the aging population.

## 1 Introduction

In recent years, population aging has emerged as a significant global demographic trend (1, 2). The global aging phenomenon has introduced unprecedented challenges, particularly exacerbating issues related to cognitive impairment (3). Cognitive impairment involves the reception and processing of information, characterized by memory loss, decreased understanding, impaired attention, and calculation difficulties (4). This cognitive decline is regarded as the preclinical stage of Alzheimer’s disease, potentially influenced by lifestyle, physical function, and other factors (5). China has the largest older adult’s population in the world. In 2015, the older adult’s population was 201 million, projected to increase to 479 million by 2050 (6). Concurrently, the prevalence of dementia among the older adults in China has surged. A 2018 national survey estimated that 15 million older adults’ individuals in China were afflicted with dementia (3). Given the limited efficacy of curative treatments for dementia, prevention is crucial (7). Cognitive decline is considered a primary risk factor for neurodegenerative diseases such as dementia (8), underscoring the urgency of identifying modifiable risk factors (9).

As the prevalence of Alzheimer’s disease increases, China is experiencing a rapid digital transformation, with expanding Internet penetration affecting more aspects of people’s lives. Internet use not only facilitates daily convenience but also enhances social communication, potentially improving quality of life. Compared to developed countries, the educational level of China’s older adults’ population is relatively lower, which may limit their use of new technologies and smart devices (10–12). According to the 53rd China Internet Development Statistical Report in 2023, there are 317 million non-internet users in China (13). Of these, 39.8% are individuals over 60 years old. A significant portion of the older adults risks digital exclusion, which could severely impact their quality of life (14).

Theoretically, the use of the Internet and mobile Internet tools can simplify daily activities for the older adults, such as online shopping and hailing taxis. Additionally, mobile Internet usage enhances interpersonal communication, offers more efficient access to external information, and may slow cognitive decline and reduce the incidence of dementia among the older adults. To empirically examine the impact of Internet use on the cognitive abilities of the older adults in China, this study utilizes data from the China Health and Pension Follow-up Survey (CHARLS 2020). It employs correlation analysis, stratified regression, and multiple linear regression to assess how Internet usage across various regions influences cognitive functions among the older adults.

With the aggravation of aging and the rapid popularization of digital technology, academic circles began to pay attention to the potential impact of Internet use on the life and health of the older adults, especially its role in the protection of cognitive function. Existing studies have explored the challenges under the background of global aging, the impact of the Internet on the quality of life of the older adults, and the relationship between Internet use and cognitive function, but there are still problems such as limited samples, single research perspective and insufficient variable control. These studies provide an important theoretical basis and direction for this study, but also highlight the research gaps that need to be further explored.

The importance of this study lies not only in the breakthrough in academic field, but also in its practical application value. By systematically analyzing the influence path of Internet use on the cognitive function of the older adults, the research results will provide scientific basis for formulating public policies to promote digital inclusion and improve their cognitive health. This is of great guiding significance for coping with the complex challenges brought by an aging society, especially in China, a country with the largest older adults’ population in the world.

In a word, this paper aims to fill the gaps in the current literature and provide new theoretical perspectives and policy suggestions through empirical analysis of the impact of Internet use on the cognitive function of the older adults. The research results not only deepen the understanding of the relationship between Internet and cognitive health, but also provide an important reference for public health decision-making in an aging society.

## 2 Literature review

### 2.1 Challenges and trends of global aging

Global population aging is rapidly becoming an important social and economic issue in the 21st century. Aging not only brings great pressure to public health system, but also affects the sustainable development of family structure and labor market. At the same time, the cognitive decline of the older adult’s group is increasingly prominent, which poses new challenges to social well-being and medical resources (15).

Previous studies have shown that the decline of cognitive function is an early manifestation of neurodegenerative diseases, such as Alzheimer’s disease. Although there is no effective treatment at present, lifestyle intervention is considered as an important way to delay cognitive decline (16). In this context, the popularity of the Internet provides a new opportunity to solve this problem. Internet use may become a potential intervention to improve the cognitive health of the older adults by promoting social participation, information acquisition and stimulation of brain activity (17). In addition, there are significant differences in the aging process and technology acceptance in different countries and regions. For example, the Internet usage rate of the older adults in developed countries is generally high, while the Internet penetration rate of the older adults in developing countries (such as China) is still low, which leads to the risk of digital exclusion for some older adults’ groups (18). This digital exclusion may further aggravate its social isolation and cognitive function degradation.

Therefore, it is of great practical significance to study the influence of Internet use on the cognitive function of the older adults, especially in countries with rapid aging and increasing digitalization, such as China. This can not only provide theoretical basis for delaying cognitive decline, but also provide empirical support for promoting the formulation of digital inclusion policies for the older adults.

### 2.2 The impact of internet on the life of the older adults

Research by Wang and Chen (19) indicates that Internet usage significantly enhances social participation among the older adults, positively impacting their health through increased social interactions, which are crucial for their physical and mental well-being. According to Aggarwal et al. (20), the advent of the Internet enables the older adults to engage in online shopping and manage business activities. Physical limitations may prevent some older adults’ individuals from performing certain activities; however, the Internet facilitates these tasks for them. Internet use among the older adults facilitates access to information, helps establish social connections, and positively impacts their quality of life. Zhou et al. (21) recommend moderate Internet use among the older adults, noting that excessive use may increase depression symptoms and cognitive decline, whereas moderate use can enhance mental health and alleviate loneliness.

### 2.3 Research on the relationship between cognitive ability and internet use

Research by Samantha Dequanter et al. (22) indicates that as cognitive vulnerability increases, the frequency and diversity of Internet use decrease, particularly among those with severe cognitive impairments. As cognitive vulnerability rises, Internet usage declines, influenced by factors such as age, income, living conditions, and broader biopsychosocial characteristics. Van Der Wardt et al. (23) highlighted the complexities of technology adoption among the older adults, considering factors like dementia and depression, and concluded that ICT usage by the older adults enhances cognitive engagement and emotional well-being in those with cognitive impairments. Zhang and Zhou (24) investigated how Internet use, through activities like physical exercise, entertainment, and study, can prevent cognitive decline, based on data from a family follow-up survey in China. This research identified a positive correlation between Internet use and the cognitive abilities of the older adults. The findings advocate for policy measures to enhance Internet skills and encourage active participation in leisure activities to promote cognitive health among the older adults.

Although the existing research has extensively discussed the relationship between aging and cognitive decline, some cutting-edge research also involves the positive impact of Internet use on the lives of the older adults. However, there are still several important limitations in the research of related fields. Firstly, the research perspective is relatively single. Many studies mainly focus on the influence of internet use on the physical health or mental health of the older adults, but there is still a lack of systematic research on the mechanism of internet use in cognitive function (25, 26). Secondly, the research sample has limitations. Some studies are based on specific regions or small-scale samples, lacking national representativeness, which limits the universality of research conclusions (18, 27). Thirdly, insufficient variable control is also a prominent problem in existing research. Some studies fail to fully consider multiple influencing factors such as education level, income status and health status, which may lead to the deviation of research conclusions (17).

In view of the above limitations, this study has important academic and practical value. On the one hand, this paper aims to make up for the sample limitations of previous studies and provide a more comprehensive empirical analysis (28) by using the data of the nationwide representative China Health and Pension Follow-up Survey (CHARLS 2020). Although the data of CHARLS database has been widely used and a large number of articles have been published, the advantages of national representation and data universality of CHARLS database are still undeniable. In addition, this paper uses the cross-sectional data of CHARLS 2020, which is the latest available data set. Since its release in 2023, the data circulation time is not long; On the other hand, by introducing multivariable control, this study systematically discusses the influence path of Internet use on the cognitive function of the older adults, deepens the theoretical understanding of this field, and avoids the deviation of the research conclusions as much as possible (29).

## 3 Materials and methods

### 3.1 Data source and sample

The data for this study were sourced from the 2020 China Health and Retirement Longitudinal Study (CHARLS 2020) (28). CHARLS, conducted by the National Development Research Institute of Peking University, is a nationally representative survey encompassing 450 villages across 28 provinces, including municipalities and autonomous regions. The CHARLS questionnaire collects extensive personal and family data on middle-aged and older adults’ individuals, including variables related to physical and mental health (e.g., depressive symptoms, cognitive ability, and self-rated health), lifestyle habits (e.g., exercise, Internet usage, and sleep), and demographics (e.g., gender, age, marital status, and education level). This dataset provides substantial support for investigating the causal relationship between Internet use and cognitive ability among the older adults in China. Detailed information about the CHARLS dataset is available on its website at http://charls.pku.edu.cn/ (accessed October 13, 2024).

The 2020 CHARLS data selected for this study represents the most recent dataset, covering 19,395 respondents. In alignment with the study’s objectives, respondents under 60 years old and those with missing key variables were excluded, resulting in a total sample of 7,142, comprising 3,991 males and 3,151 females. All participants provided informed consent prior to the interview. The data collection for CHARLS was approved by the Peking University Biomedical Ethics Review Committee (approval number: IRB00001052-11015).

### 3.2 Variable selection

#### 3.2.1 Independent variable

In this study, Internet usage is designated as the independent variable. The CHARLS2020 questionnaire measures Internet usage with questions including: (1) “Did you surf the Internet in the past month?,” (2) “Do you make payments via mobile phone?,” (3) “Do you use WeChat?,” and (4) “Can you send a WeChat circle of friends?” Respondents must answer “yes” or “no” to these questions, with answers recorded as binary variables: “yes” as “1” and “no” as “0.” These questions are sequentially dependent; respondents proceed to questions 2 and 3 only if they answer “yes” to question 1, and to question 4 only if they answer “yes” to question 3. Each “yes” response is scored, with total scores ranging from 0 to 4. A higher score indicates greater Internet usage.

#### 3.2.2 Dependent variable

This study identifies cognitive ability level as the dependent variable. Through face-to-face interviews and surveys, CHARLS 2020 investigators assessed three cognitive functions: memory, orientation, attention, and visual–spatial ability. During the memory test, investigators read aloud 10 Chinese nouns slowly and then asked respondents to recall as many words as possible. Memory ability was scored based on the average number of words recalled immediately and again after 4 min, with scores ranging from 0 to 10. Orientation and attention were evaluated using the Mini Mental State Examination (MMSE) (30), which includes tasks such as serial subtraction of 7, identifying the date, day of the week, and season, with scores derived from the sum of correct answers. Visual–spatial ability was assessed through a drawing task where participants replicated an image of two overlapping pentagons, earning 1 point for successful and 0 points for unsuccessful attempts. The overall cognitive score, ranging from 0 to 21, was calculated by summing the scores from memory, orientation, attention, and visual–spatial tasks. Lower scores indicate poorer cognitive abilities.

To ensure the appropriateness of the MMSE for our Chinese older adult cohort, we have considered both the validity and reliability of the MMSE within this specific population. Recent studies utilizing large, representative samples from the Chinese Health and Retirement Longitudinal Study (CHARLS) have confirmed the three-dimensional factor structure of the MMSE, supporting its theoretical framework in the Chinese context. The total and subscale scores of the MMSE have shown acceptable model-data fit, demonstrating its capacity to accurately assess cognitive functions among older Chinese adults. Notably, the reliability of the MMSE total scores was estimated at 0.78, indicating moderate measurement precision within this demographic group (31).

Furthermore, item response theory (IRT) analysis has revealed differential functioning of certain MMSE items across various age and education level subgroups (32). This finding suggests the need for careful consideration when applying the MMSE to ensure accurate measurement of cognitive abilities across the diverse educational backgrounds prevalent among older adults in China.

By leveraging these insights, our study employs the MMSE with a nuanced understanding of its psychometric properties tailored to the Chinese older adult population, enhancing the rigor and relevance of our cognitive assessments.

#### 3.2.3 Control variable

To elucidate the significant influence of independent variables on dependent variables, this study controlled for demographic covariates potentially related to cognitive ability levels in CHARLS. Demographic data collected included gender, age categories (60–69, 70–79, 80–89, and 90+ years), educational attainment (illiteracy, primary, junior high, and senior high and above), marital status (married, other), and residence (rural, urban).

### 3.3 Statistical analysis method

**Figure 1:**
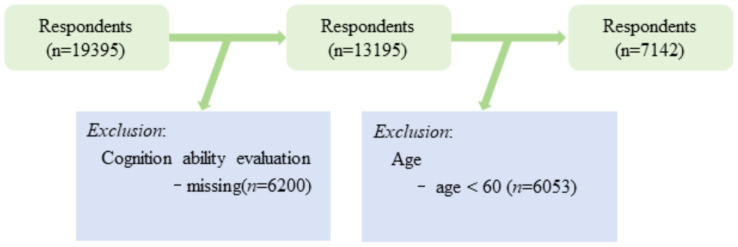
Sample screening process.

In this study, Stata/MP 18.0 and SPSS 24.0 (Armonk, NY: IBM Corp.) were utilized for data screening, cleaning, and analysis. Initially, the study filtered the CHARLS2020 dataset to exclude individuals under 60 years and those with missing key variables (Figure 1). This resulted in a total sample size of 7,142. Subsequently, the study described the composition of the effective sample. Correlation analysis was then conducted to examine the relationships between each variable and cognitive levels. Hierarchical regression analysis was performed with cognitive level as the dependent variable and Internet usage as the independent variable, incrementally including different control variables to elucidate their impacts. To further investigate the impact of Internet usage on older adults’ cognitive abilities, the study utilized multiple linear regression analysis based on four Internet usage indicators. Due to the progressive relationship among the four variables, including them simultaneously in the model introduces collinearity. Consequently, four separate regression equations were employed to delineate the varying degrees of Internet use’s impact on older adults’ cognitive levels (33).

## 4 Analysis

### 4.1 Sample description

In this study, individuals aged over 60 years from the CHARLS2020 dataset were selected as the subjects. Following data cleansing and screening processes, respondents under 60 years old and those with missing key variables were excluded. Consequently, the study comprised a total of 7,142 valid participants, including 3,991 males and 3,151 females. Table 1 presents the basic demographic structure of these valid samples.

**Table 1:** Distribution of valid survey samples.

Variable		Number/cases	Percentage/%
Gender	Male	3,991	55.88
	Female	3,151	44.12
Age	60–69 years old (Inclusive)	4,376	61.27
	70–79 years old (Inclusive)	2,347	32.86
	80–89 years old (Inclusive)	404	5.66
	90 years old and above	15	0.21
Degree of Education	Below primary school	2,683	37.57
	Primary school	1794	25.12
	Middle school	1,565	21.91
	Above Middle school	1,100	15.40
Marital Status	Not in marriage	1,214	17.00
	Married	5,928	83.00
Residence	Rural	3,951	55.32
	Urban	3,191	44.68
Use Internet	Yes	2,293	32.11
	No	4,849	67.89
Use Wechat (*n* = 2,293)	Yes	1992	86.87
	No	301	13.13
Use Mobile Payment (*n* = 2,293)	Yes	1,118	48.76
	No	1,175	51.24
Use Wechat Moment (*n* = 1992)	Yes	1,174	58.94
	No	818	41.06

Descriptive statistics indicate that only 32.11% of the older adults over 60 years old utilize the internet. Within this demographic, a significant majority (86.87%) use WeChat, demonstrating high engagement with this application. Conversely, adoption rates for mobile payment (48.76%) and WeChat’s ‘Friends Circle’ (58.94%) are comparatively lower. In the context of the mobile internet era, the data show that only 32.11% of the older adults engage with the internet, 27.89% use WeChat for communication, and a mere 16.44% proficiently share content on WeChat’s ‘Friends Circle.’ The penetration rate for mobile payment tools is notably low, with only 15.65% of the older adults effectively using these services. This data suggests widespread digital exclusion among the older adults, particularly in proficient use of mobile internet tools. A potential contributor to this digital divide is the education level of the older adult’s population, which is generally low. Notably, China implemented compulsory nine-year education starting in 1986, and all participants in this sample were born before this policy was enacted, leading to an average education duration of only 4.2 years (34).

**Figure 2:**
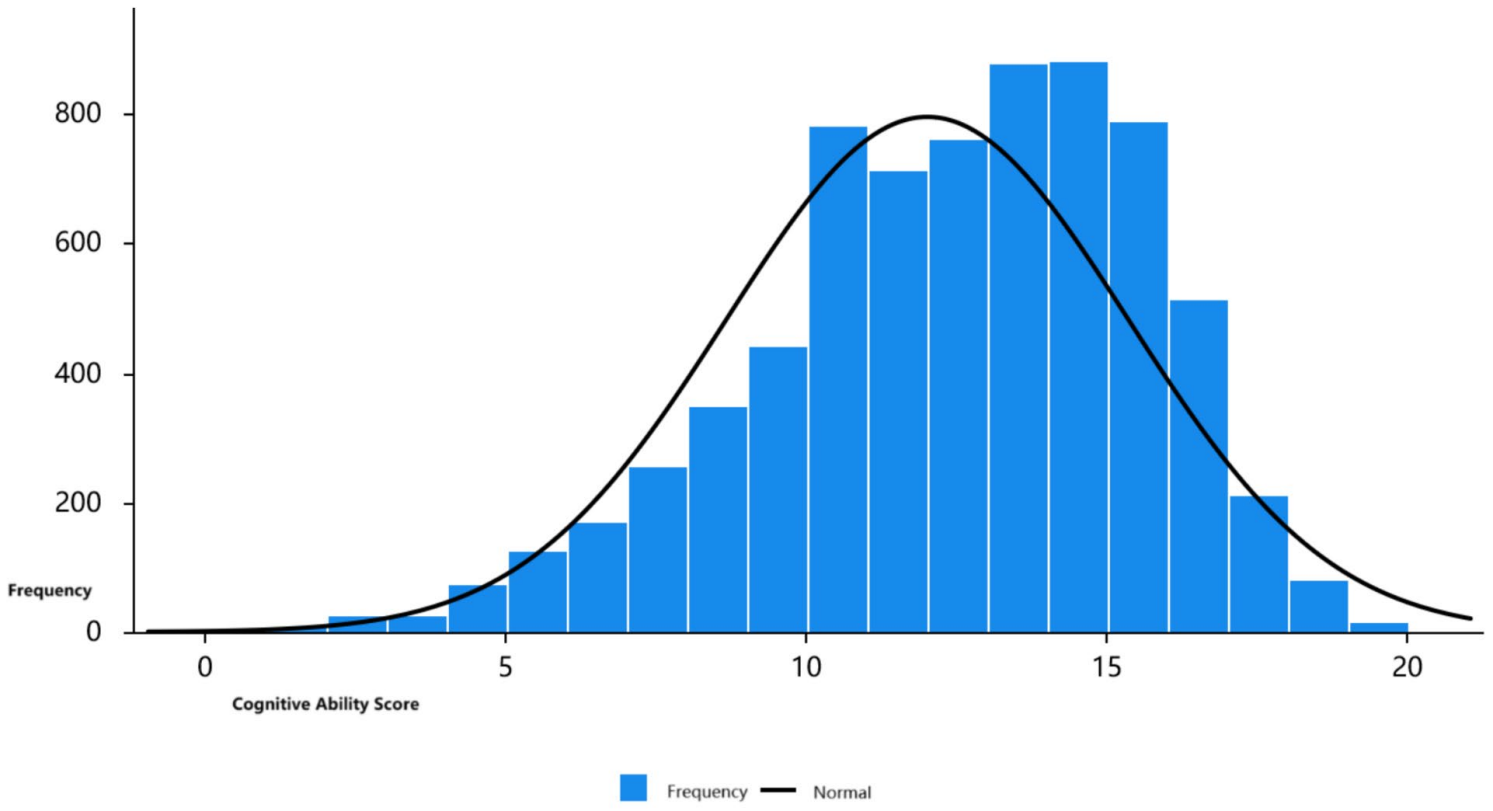
Distribution of cognitive abilities scores.

In this study, respondents’ cognitive levels were assessed across three domains: memory, orientation and attention, and spatial vision ability. The overall cognitive ability score, which ranges from 0 to 21, inversely reflects cognitive performance—the lower the score, the poorer the cognitive ability. Figure 2 displays the cognitive ability scores for all 7,142 respondents.

### 4.2 Correlation analysis

Spearman correlation analysis is a non-parametric statistical method used to assess the correlation between two variables (35–37). It evaluates the monotonic relationship between the ranks of two datasets. Unlike the Pearson correlation coefficient, which primarily detects linear relationships, Spearman’s correlation is capable of identifying more general monotonic relationships—namely, it can detect consistent increases or decreases in one variable in relation to another.

In this study, correlation analysis was employed to investigate the relationships between internet usage, WeChat use, mobile payment adoption, engagement with WeChat’s Friends Circle, and demographic variables such as gender and age, with cognitive ability levels. The strength of these correlations was quantified using Spearman correlation coefficients. Detailed results of this analysis are presented in Table 2.

**Table 2:** Spearman correlation analysis results.

		Cognitive abilities
Use Internet	Correlation coefficient	0.317***
	*p*-value	<0.001***
Use Wechat	Correlation coefficient	0.133***
	*p*-value	<0.001***
Use Mobile Payment	Correlation coefficient	0.192***
	*p*-value	<0.001***
Use Wechat Moment	Correlation coefficient	0.188***
	*p*-value	<0.001***
Gender	Correlation coefficient	0.110***
	*p*-value	<0.001***
Age	Correlation coefficient	−0.098***
	*p*-value	<0.001***
Degree of Education	Correlation coefficient	0.440***
	*p*-value	<0.001***
Marital Status	Correlation coefficient	0.113***
	*p*-value	<0.001***
Residence	Correlation coefficient	0.205***
	*p*-value	<0.001***

*** represents the significance level of 1%.

### 4.3 Regression analysis

Hierarchical regression analysis is a flexible method that allows researchers to control the sequence in which variables are introduced into the regression model, typically employed to test theories or hypotheses regarding variable relationships (38–40). The results from Model 1 reveal a significant positive correlation between the extent of Internet use and cognitive ability levels, indicating that higher Internet usage correlates with higher cognitive ability. Subsequent models, which incorporate social characteristics like residence and education level, as well as personal characteristics such as gender, age, and marital status, continue to show significant associations with cognitive levels. Specifically, Model 2 and Model 3 demonstrate that living in urban areas, higher educational attainment, male gender, being married, and younger age are all associated with higher cognitive abilities. The findings of this stratified regression are detailed in Table 3.

**Table 3:** Hierarchical regression analysis results.

	Cognitive abilities		
	Model 1	Model 2	Model 3
Evaluate Internet Use	0.757***(0.026)	0.390***(0.027)	0.357***(0.027)
Residence		0.557***(0.073)	0.657***(0.074)
Degree of Education		1.046***(0.036)	0.993***(0.037)
Gender			0.279***(0.074)
Marital Status			0.522***(0.097)
Age			−0.288***(0.060)
Cons	11.317***(0.044)	10.202***(0.056)	9.788***(0.106)
*N*	7,142	7,142	7,142
Adjust *R*^2^	0.109	0.220	0.228

*** represents the significance level of 1%. Values in brackets are standard errors.

The four independent variables quantifying Internet usage reflect the proficiency and depth of Internet engagement. The hierarchy of usage starts with the most prevalent, Internet browsing, followed by WeChat usage, interacting within WeChat’s Friends Circle, and finally mobile payment usage. Given the progressive relationship among these indicators, conducting separate regression analyses is crucial to effectively present the research findings. Initially, this study applied multiple linear regression (41–43) to assess how varying degrees of Internet use affect the cognitive levels of the older adults, with the results detailed in Table 4. The regression analysis indicates that Internet usage significantly enhances the cognitive abilities of the older adults. Among the control variables, age and gender did not significantly impact the assessment of Internet proficiency and its breadth on cognitive ability. Conversely, being married is associated with higher cognitive levels compared to being unmarried, and higher educational attainment correlates with improved cognitive function. These findings reaffirm that cognitive abilities of individuals living in rural areas are significantly lower than those in urban settings.

**Table 4:** Multiple regression analysis results.

	Cognitive abilities			
	Model 4	Model 5	Model 6	Model 7
Use Internet	1.041***(0.083)			
Use Wechat		0.772***(0.159)		
Use Mobile Payment			0.502***(0.112)	
Use Wechat Moment				0.526***(0.116)
Gender	0.276***(0.074)	−0.017 (0.112)	−0.094 (0.113)	−0.096 (0.116)
Marital Status	0.527***(0.097)	0.433***(0.166)	0.432***(0.166)	0.592***(0.173)
Residence	0.687***(0.074)	0.684***(0.115)	0.659***(0.115)	0.738***(0.121)
Age	−0.291***(0.060)	0.170 (0.115)	0.219 (0.116)	0.208 (0.120)
Degree of Education	1.020***(0.036)	0.816***(0.052)	0.789***(0.053)	0.774***(0.055)
Cons	9.737***(0.106)	10.601***(0.223)	11.125***(0.184)	11.004***(0.195)
*N*	7,142	2,293	2,293	1992
Adjust *R*^2^	0.227	0.163	0.162	0.172
D-W value	1.886	1.904	1.897	1.899

*** represents the significance level of 1%. Values in brackets are standard errors.

Empirical results indicate a significant association between Internet use and cognitive ability among the older adults, with other demographic factors also influencing cognitive performance to varying degrees. Hierarchical regression analysis confirms that Internet use is positively correlated with cognitive ability, supporting the cognitive reserve theory. This theory posits that engaging in intellectually stimulating activities via the Internet can enhance or maintain cognitive functions, potentially slowing cognitive decline and preventing Alzheimer’s disease. Additionally, the analysis identifies place of residence and educational background as significant demographic influencers of cognitive ability. Urban residents typically exhibit higher cognitive abilities than their rural counterparts, likely due to denser population interactions and broader social activities, which positively affect health and cognitive function (44). Furthermore, higher educational levels correspond to improved cognitive abilities, aligning with the cognitive reserve theory, which suggests that individuals with more education possess greater cognitive reserves. This ability enables the brain to adapt and find alternative solutions to challenges, such as aging or injury. The study also notes that gender, age, and marital status influence cognitive ability, with men and married individuals displaying higher cognitive levels, and younger individuals showing better cognitive performance.

Multiple linear regression analysis demonstrates that Internet use significantly enhances the cognitive abilities of the older adults and helps prevent depression. Notably, the cognitive benefits increase with the use of mobile Internet and mobile payment technologies. In the context of digitalization and aging, promoting digital inclusion for the older adults through targeted policies can positively impact their mental health and cognitive function. Despite these findings, digital exclusion remains prevalent among the older adults in China (45). Encouraging the use of the Internet through community and volunteer activities can increase older adults’ engagement with online platforms. Furthermore, efficient public services can mitigate the adverse effects of digital exclusion on the cognitive abilities and overall quality of life of the older adults.

## 5 Discussion

This study investigates the impact of internet use on cognitive functions among the older adults in China, drawing on data from the China Health and Retirement Longitudinal Study (CHARLS 2020). The sample included 7,142 individuals aged 60 and above, covering 450 villages across 28 provinces in China. Through correlation analysis and hierarchical regression, this research revealed a significant positive relationship between internet use and cognitive functions among the older adults.

This study found that the use of the Internet has a significant positive impact on the cognitive function of the older adults. This result shows that the Internet can provide protection for the older adults through information acquisition, social interaction and cognitive stimulation, thus slowing down the decline of cognitive ability. These findings are consistent with previous studies. For example, Yu and Fiebig (46) showed that Internet use can be used as a protective factor to prevent cognitive decline in middle-aged and older adults’ people. Xia et al. (47) show that Internet use has a significant positive impact on cognitive function and supports the maintenance of cognitive ability of the older adults. In addition, this study also shows that the effects of Internet use are different in different regions and different population characteristics, which provides a new perspective for understanding the influence mechanism of Internet use.

The research results of this paper not only support the conclusions of existing literature, but also expand the research in this field to some extent. For example, Choi and DiNitto (18) pointed out that Internet use has a positive impact on cognitive health by promoting social contact and reducing loneliness. However, unlike some studies that mainly focus on mental health, this paper pays more attention to how Internet use directly affects cognitive function. This expansion makes up for the deficiency of the current literature on the mechanism of Internet use. In contrast, some studies show that the use of the Internet may lead to adverse effects. For example, Yu et al. (48) found that using the Internet for business-related activities less than once a week is related to poor cognitive function. Kim and Han (49) found that for the older adults born in 1941 or before, the adverse impact of the transition to Internet use is more serious. The research of this paper emphasizes the importance of moderate use and points out the risks that may be brought by excessive dependence on the Internet.

Theoretically, this study deepens the understanding of the relationship between Internet use and cognitive reserve. Cognitive reserve theory holds that the level of individual cognitive activity can improve cognitive health by enhancing neuroplasticity (50). Internet use, especially online learning and social interaction, may delay the decline of cognitive ability related to aging by stimulating cognitive processing. This discovery extends the applicability of cognitive reserve theory in the older adults’ population.

This study is based on the data of the China Health and Pension Follow-up Survey (CHARLS 2020), which is one of the most representative and widely used data sets to study the health and pension problems of middle-aged and older adults’ people in China. A large number of studies use CHARLS data to explore the health status, social participation and cognitive function of the older adults. For example, Li et al. (51) analyzed the relationship between social integration and cognitive function trajectory of the older adults by using the data of CHARLS, and the results showed that social integration significantly affected the cognitive function development trajectory of the older adults. Mose et al. (52) used CHARLS data to focus on the influence of social activities on cognitive function, and further emphasized the important role of social participation in maintaining cognitive health. The data provided by Zhou et al. (53) interest CHARLS explored the significant correlation between the types, frequencies and diversity of social participation and the cognitive function of the older adults, and provided insight into the influence of different forms of social participation on cognitive outcomes. By using the latest data of CHARLS 2020, this paper not only verifies the positive influence of Internet use on cognitive function, but also improves the robustness of the results by combining multivariable control methods. The advantage of these data is that they contain multi-dimensional information of the older adults (such as health status, economic conditions, social participation, etc.), which provides the possibility for analyzing the complex mechanism of Internet use. In addition, compared with other data based on small-scale samples or regional studies, the national representativeness and timeliness of CHARLS data provide a solid foundation for the universality of this research result.

## 6 Conclusion

This study confirmed a significant positive correlation between internet usage and the enhancement of cognitive abilities among older adults in China. The findings illustrate that internet usage notably improves overall cognitive scores, particularly enhancing memory and executive functions in the older adults. Moreover, the benefits of internet usage are more pronounced among older adults with higher educational levels and those residing in urban areas, suggesting that socio-economic factors play a crucial role in digital engagement outcomes. This study underscores the importance of implementing policies to promote digital inclusivity, supporting cognitive health and social participation among the older adults.

These conclusions provide a basis for policymakers to develop strategies that enhance digital skills and internet access among the older adults, thereby improving their quality of life and delaying cognitive decline. Our research calls for more societal and technological interventions to mitigate the challenges brought about by global aging.

## Data Availability

The datasets presented in this study can be found in online repositories. The names of the repository/repositories and accession number(s) can be found in the article/supplementary material.
